# The complementary role of MRI and FET PET in high-grade gliomas to differentiate recurrence from radionecrosis

**DOI:** 10.3389/fnume.2023.1040998

**Published:** 2023-04-27

**Authors:** Arpita Sahu, Ronny Mathew, Renuka Ashtekar, Archya Dasgupta, Ameya Puranik, Abhishek Mahajan, Amit Janu, Amitkumar Choudhari, Subhash Desai, Nandakumar G. Patnam, Abhishek Chatterjee, Vijay Patil, Nandini Menon, Yash Jain, Venkatesh Rangarajan, Indraja Dev, Sridhar Epari, Ayushi Sahay, Prakash Shetty, Jayant Goda, Aliasgar Moiyadi, Tejpal Gupta

**Affiliations:** ^1^ Department of Radiodiagnosis, Tata Memorial Hospital and Homi Bhabha National Institute, Mumbai, India; ^2^ Department of Radiation Oncology, Tata Memorial Hospital and Homi Bhabha National Institute, Mumbai, India; ^3^ Department of Nuclear Medicine, Tata Memorial Hospital and Homi Bhabha National Institute, Mumbai, India; ^4^ Department of Radiology, The Clatterbridge Cancer Centre NHS Foundation Trust, Pembroke Place, Liverpool, United Kingdom; ^5^ Department of Medical Oncology, Tata Memorial Hospital, Homi Bhabha National Institute, Mumbai, India; ^6^ Department of Pathology, Tata Memorial Hospital and Homi Bhabha National Institute, Mumbai, India; ^7^ Department of Neurosurgery, Tata Memorial Hospital and Homi Bhabha National Institute, Mumbai, India

**Keywords:** high-grade glioma, glioblastoma, MRI, PET, radionecrosis, recurrence, biological imaging

## Abstract

**Introduction:**

Conventional magnetic resonance imaging (MRI) has limitations in differentiating tumor recurrence (TR) from radionecrosis (RN) in high-grade gliomas (HGG), which can present with morphologically similar appearances. Multiparametric advanced MR sequences and Positron Emission Tomography (PET) with amino acid tracers can aid in diagnosing tumor metabolism. The role of both modalities on an individual basis and combined performances were investigated in the current study.

**Materials and Methods:**

Patients with HGG with MRI and PET within three weeks were included in the retrospective analysis. The multiparametric MRI included T1-contrast, T2-weighted sequences, perfusion, diffusion, and spectroscopy. MRI was interpreted by a neuroradiologist without using information from PET imaging. 18F-Fluoroethyl-Tyrosine (FET) uptake was calculated from the areas of maximum enhancement/suspicion, which was assessed by a nuclear medicine physician (having access to MRI to determine tumor-to-white matter ratio over a specific region). A definitive diagnosis of TR or RN was made based on the combination of multidisciplinary joint clinic decisions, histopathological examination, and clinic-radiological follow-up as applicable.

**Results:**

62 patients were included in the study between July 2018 and August 2021. The histology during initial diagnosis was glioblastoma, oligodendroglioma, and astrocytoma in 43, 7, and 6 patients, respectively, while in 6, no definitive histological characterization was available. The median time from radiation (RT) was 23 months. 46 and 16 patients had TR and RN recurrence, respectively. Sensitivity, specificity, and accuracy using MRI were 98, 77, and 94%, respectively. Using PET imaging with T/W cut-off of 2.65, sensitivity, specificity, and accuracy were 79, 84, and 80%, respectively. The best results were obtained using both imaging combined with sensitivity, specificity, and accuracy of 98, 100, and 98%, respectively.

**Conclusion:**

Combined imaging with MRI and FET-PET offers multiparametric assessment of glioma recurrence that is correlative and complimentary, with higher accuracy and clinical value.

## Introduction

Gliomas are classified based on their molecular characteristics in the 2021 WHO Classification of Tumors of the Central Nervous System (1). High-grade gliomas (HGG) account for 14% of all tumors and 49% of malignant tumors (2). Glioblastoma multiforme (GBM) is the most common malignant type of primary astrocytomas, associated with poor prognosis with median survival in the range of 15–18 months from contemporary clinical trials (3, 4). The survival of grade 3 astrocytoma and oligodendrogliomas are better, in the range of 10–14 years (5). The standard treatment in HGG includes maximal safe resection followed by adjuvant chemoradiation and chemotherapy (6–8). Disease recurrence is encountered during or a few months after completion of maintenance chemotherapy in GBM depicting aggressive tumor biology, or after several years of treatment completion in IDH-mutant gliomas. Also, following radiation (RT), treatment-related changes in the form of radionecrosis (RN) can be encountered in a proportion of patients, which can mimic progressive disease (9–11). The spectrum of RN ranges from asymptomatic to severe neurological worsening, with the majority responding to medical decompressive therapy, with some refractory cases requiring surgery or anti-angiogenic therapy with Bevacizumab (12, 13). According to the Response Assessment in Neuro-Oncology (RANO) criteria, FLAIR/T2 hyperintensity is used as a stand-in for the tumor's nonenhancing component. When compared to the Macdonald and RECIST criteria, the RANO was just as effective at spotting radiological progression before clinical deterioration (14–18).

Magnetic resonance imaging (MRI) forms an integral role in the management of brain tumors aiding in diagnosis, treatment planning, response evaluation, and surveillance (19, 20). On conventional MRI sequences, accurate diagnosis of disease recurrence can be challenging since treatment-related changes can often present with similar morphological appearances (21). The use of advanced MR sequences in the form of perfusion, arterial spin labeling (ASL), and MR spectroscopy (MRS) can be of particular interest to differentiate recurrence from RN due to different underlying pathological processes, which otherwise can present with a morphologically similar appearance on conventional sequences (22, 23).

The role of positron emission tomography (PET) is emerging in brain imaging, aiding in the identification of different tumor histologies or differentiating treatment-related changes post-therapy from disease recurrence (24). The uptake of amino acid tracers is independent of the integrity of the blood-brain barrier, hence the evaluation of non-enhancing gliomas can be done with amino acid PET (25–27). Among amino acid tracers, O-[2-(18F)-fluoroethyl]-L-tyrosine (FET) has become the most widely used radiotracer for brain tumor diagnostics (28, 29). Since tumor-induced metabolism is reflected by cellular proliferation utilizing amino acids, the use of FET-PET can potentially differentiate tumor progression from RN which is an inflammatory process. Therefore, in conjunction with MRI, PET can provide more accurate diagnosis especially in clinically equivocal situations (30, 31).

This study compares imaging features of MRI with amino acid PET tracer O-(2-[18F] fluoroethyl-L-tyrosine (FET) to differentiate tumor recurrence from radionecrosis in high-grade gliomas.

## Materials and methods

### Patient selection

The current study was a retrospective analysis at a tertiary care cancer center. The study was conducted after clearance from the Institutional Ethics Committee (IEC), and a waiver for obtaining informed consent was granted. Patients with histologically proven HGG (grade 3 astrocytoma or oligodendrogliomas, GBM) during index presentation were included in the study. All patients were treated with maximal safe resection followed by adjuvant RT with concurrent temozolomide followed by adjuvant temozolomide as per standard institutional practice. During the adjuvant treatment and after completion, patients were followed up with MRI in regular intervals of 6–12 months or sooner if prompted clinically. Additional PET imaging was considered in cases of newer findings on an individual basis as decided by the responsible physician following a discussion with neuroradiologists. To be considered eligible for the current study patients were required to have MRI and amino acid imaging available within 3 weeks of each other without any oncologic treatment or neurosurgical intervention (medical decompressive therapy was allowed). Patients with lower grade glioma, brainstem gliomas, non-glial tumor on histology, incomplete imaging studies, or a gap of more than three weeks between two imaging modalities (MRI and FET) were excluded.

### Instrumentation

The MRI was performed on a 1.5 Tesla, Philips Ingenia (Amsterdam, Netherlands). MR imaging sequences for complete diagnostic evaluation of the brain included an axial FLAIR sequence (TR/TE, 8,000/80 ms; TI, 2000ms; section thickness, 5 mm); a T2-weighted turbo spin-echo sequence (TR/TE, 3,000/120 ms; section thickness, 5 mm); DWI (TR/TE, 5,400/90 ms; *b* = 0, 400, 1,000 s/mm^2^); and PWI/perfusion EPI (TR/TE, 1,400/40 ms). Measurements were performed both with and without the application of a contrast agent prebolus before applying the intravenous main bolus (gadolinium-based agent, 0.1 mmol/kg bodyweight; infusion rate, 3.5 ml/s followed by 20 ml of normal saline flush). Corresponding anatomical MRI including T2- and contrast-enhanced T1-weighted images were available, as well as other sequences such as Fluid attenuation inversion recover and gradient echo. The protocols for the perfusion measurements were adapted to the scanner performance.

For FET PET, patients were injected with 5–6 mCi (185–222 GBq) of F-18-FET on the day of imaging. Dedicated static imaging of the brain was performed at 20 min post-injection using a Philips Gemini TF TOF-64 PET/CT scanner (PET crystal-LYSO). After obtaining a scout image, a plain and post-contrast CT scan of the brain was performed in the craniocaudal direction (120 kV, 250 mAs/slice, thickness-3 mm, increment-1.5 mm, pitch of 0.938, and FOV of 300 mm). PET scanning was performed immediately after CT acquisition without changing the patient's position on the scanning table.

### Image interpretation

All MRI scans were reviewed independently on a high-resolution GE multisync LCD monitor with 5 and or 12 MP resolution neuroradiologists who were blinded to the PET findings. On T2-weighted imaging, T2 intermediate to dark signal intensity areas, excluding areas of hemorrhage and necrosis, were considered suspicious for recurrence. For MR perfusion imaging, we used the automated MR Neuro Perfusion application within the Philips IntelliSpace® software toolbox. To allow for vessel exclusion and tumor margin identification, the estimated perfusion maps were coregistered and used as an overlay on anatomical MRI. ROI were placed on areas showing T2 intermediate signal which showed solid enhancement. These areas were checked on GRE to avoid bleeds. Necrosis was also avoided. ROI measuring between 30 and 50 mm^2^ were drawn at 2 to 3 places and the highest value was recorded in each case. An equally sized ROI was placed in the contralateral, normal-appearing brain tissue for calculation of the maximum rCBV (rCBVmax = CBVtumor/CBVnormal tissue).

In the case of FET PET, all reconstructed images were viewed on a display system having extended brilliance workspace software (EBW) version 4.5.3.40140, Philips Healthcare. An independent nuclear medicine physician analyzed the images, and tumor-to-contralateral white matter ratio (T/Wm) was used as a semiquantitative parameter for image interpretation. It was defined as the ratio of SUVmax of the lesion to the SUVmean of the contralateral white matter. It was calculated by placing a 3D region of interest (ROI) over the area corresponding to the suspicious area on MRI which also included all pixels above SUV max of 3.5, and another ROI over contralateral white matter, adjusted in axial, sagittal, and coronal planes. Based on prior studies, an optimum T/Wm cutoff of 2.65 was used (32).

### Outcomes analysis

The ground truth for assignment of recurrence vs. treatment changes was designated based on a multidisciplinary joint neuro-oncology meeting (JNOM) compromising of specialists from neuroradiology, nuclear medicine, neurosurgery, radiation oncology, medical oncology, and neuropathology. All the patients were individually discussed in JNOM after both imaging modalities were done, and decisions were taken based on imaging features from both MRI and PET and histopathology findings when surgery was considered. In cases of indeterminate findings, short interval imaging was considered, and final interpretation based on follow-up findings was considered as endpoints for the current analysis whenever available.

### Statistical analysis

Patient demographic data and treatment-related data were acquired from the patient's electronic medical records and radiation charts. The sensitivity, specificity, positive predictive value, negative predictive value, and accuracy were calculated for the imaging modalities both individually and combined. Descriptive statistics were used to describe the distribution of various factors across the two outcome groups, with the Pearson chi-square test or Fisher's exact test for categorical variables. All statistical analyses were performed with the SPSS software package (Version 20.0; IBM, Armonk, NewYork). For all statistical tests, a *p*-value < 0.05 was considered as a significant difference.

## Results

In the study, 62 patients were included between July 2018 and August 2021. The median age of the patients was 44 years (range: 22–74 years). Out of the total patients, 45 were male and 17 were female. Histopathology at initial diagnosis was grade 3 oligodendroglioma for 7 patients, while 6 had anaplastic astrocytoma, 43 had glioblastoma and 6 were characterized as high-grade gliomas (not further characterized). On the basis of IDH mutation, 28 cases were mutant type, 32 cases were wild type, and 2 cases had no data regarding the same. The mean interval between the last date of radiation and PET/CT or MRI scans was 23 months (range, 2–84 months). Table 1 summarizes the disease and treatment-related characteristics of the two groups.

**Table 1:** Overview of patient characteristics.

Patient characteristics (*n* = 62)	Recurrence	Radiation necrosis
Gender		
Male	38	7
Female	8	9
Tumor characteristics		
Diagnosis		
Oligodendroglioma	3	4
Astrocytoma, IDH-mutant	3	3
Glioblastoma, IDH-mutant, WHO grade IV	9	2
Glioblastoma, IDH-wild type, WHO grade IV	24	7
Glioblastoma, IDH status unknown	1	0
High-grade glioma, NOS, IDH-mutant	5	0
High-grade glioma, NOS, IDH-wild type	1	0
Molecular markers		
IDH-Mutant	19	9
IDH-Wild type	25	7
IDH Not available/ inconclusive	2	0
ATRX-Lost	13	3
ATRX-Retained	20	12
ATRX-Equivocal	2	0
ATRX-Not available	11	1
Laterality of tumor		
Right	23	6
Left	16	9
Bilateral	7	1
Treatment-related		
Radiotherapy with concurrent chemotherapy	46	16
Interval between last therapy and MRI, months, median (range)	24 months (6–84 months)	21 months (2–67 months)

**Figure 1:**
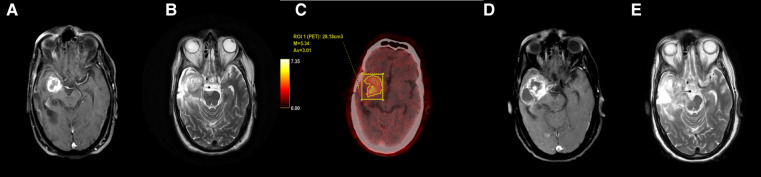
Axial T1 + Gd (**A**) and T2-weighted (**B**) images in a case of glioblastoma shows T2 intermediate signal intensity areas with thick rim enhancement. FET-PET (**C**) shows increased uptake (T/W ratio: 4.85) in the same area. Follow-up MRI (**D,E**) after 3 months showed increase in size of both solid and cystic components with likely infiltration into the cerebral peduncle. These features confirmed tumor recurrence.

**Figure 2:**
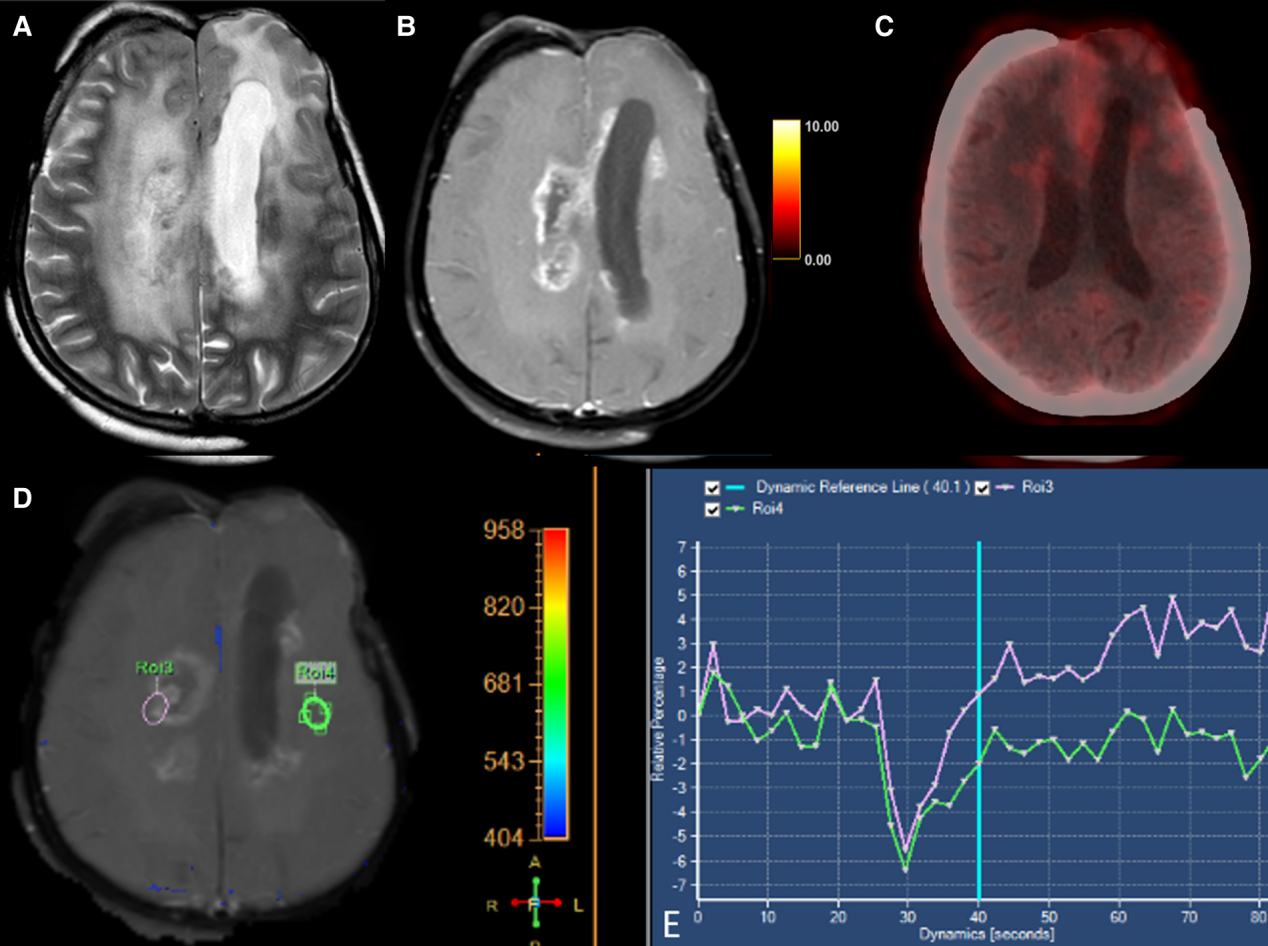
Axial T2-weighted (**A**), T1 + Gd (**B**), perfusion curve (**D,E**) and FET-PET (**C**) images in a case of grade III oligodendroglioma shows T2 hyperintense areas in the right centrum semiovale with surrounding edema. This lesion shows a “Swiss-cheese” pattern of enhancement (**B**) and hypoperfusion (**D**). FET-PET (**C**) shows no significant uptake (T/W ratio: 1.6) in the same area. These features were suggestive of radionecrosis.

**Figure 3:**
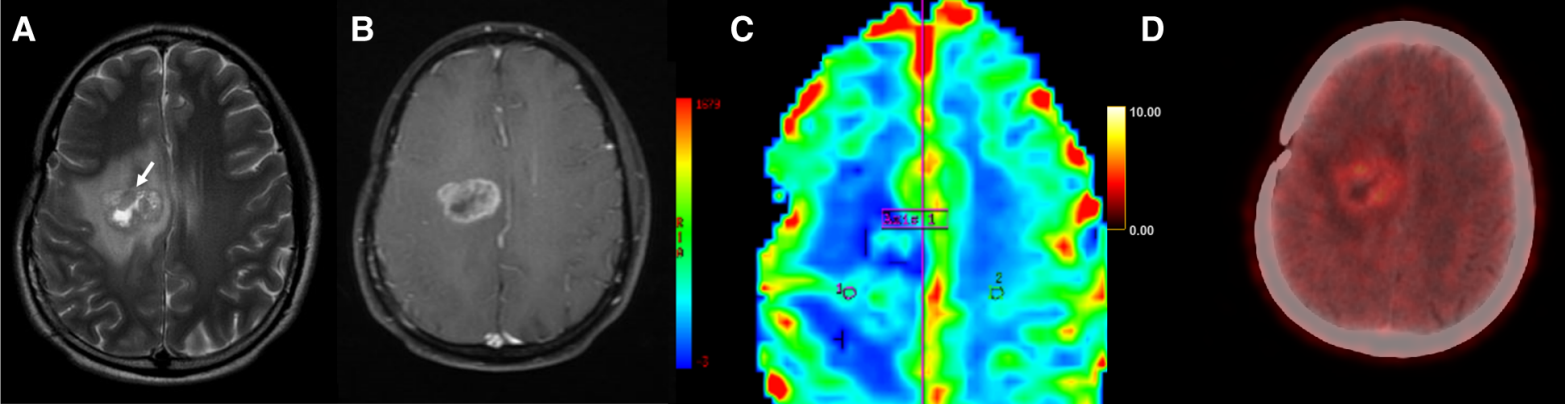
Axial T2-weighted (**A**), T1 + Gd (**B**), perfusion map (**C**) and FET-PET (**D**) images in a case of glioblastoma shows T2 intermediate areas (arrow) in the right parasagittal location with surrounding edema. Thick peripheral rim of enhancement (**B**) and hyperperfusion (**C**) is seen, with ROI1 placed in the perilesional area and ROI2 placed in normal white matter, separated by the demarcation labelled Axis 1. FET-PET (**D**) shows no significant uptake in the same area (T/W ratio: 1.9). This was a case of tumor recurrence, with false negative results on FET-PET.

**Figure 4:**
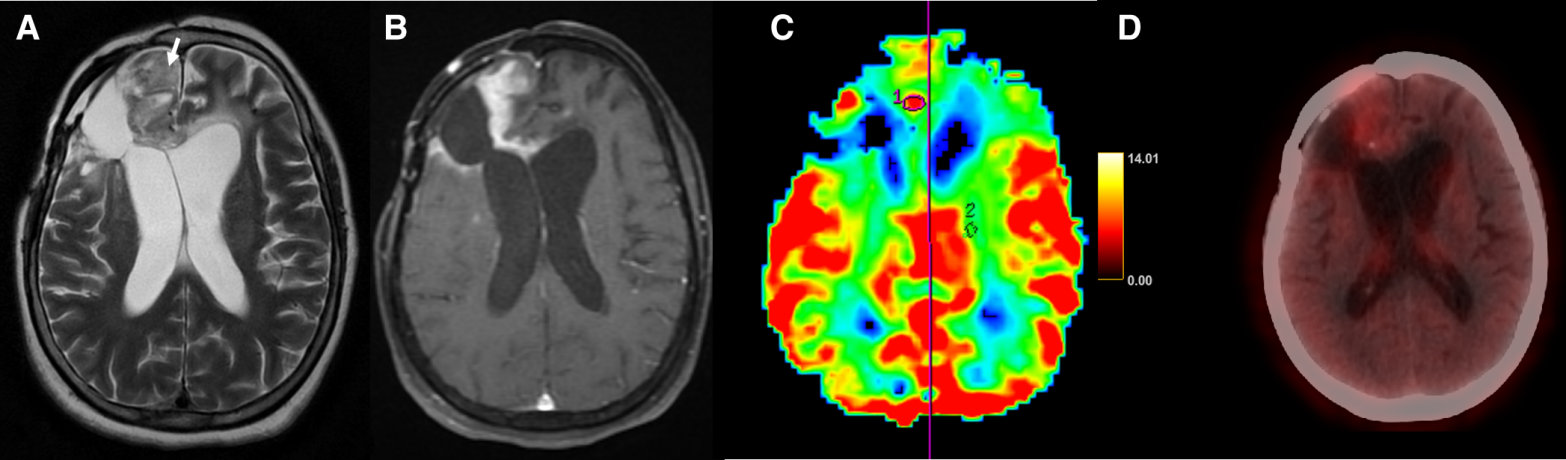
Axial T2-weighted (**A**), T1 + Gd (**B**), perfusion map (**C**) and FET-PET (**D**) images in a case of glioblastoma shows T2 intermediate areas (arrow) in the right frontal lobe. Thick peripheral enhancement (**B**) and hyperperfusion (**C**) is seen in ROI1 placed in the enhancing component and ROI2 in the normal white matter. FET-PET (**D**) shows no significant uptake (T/W ratio: 2.2) in the same area. This was a case of radionecrosis, with false positive results on MRI.

**Figure 5:**
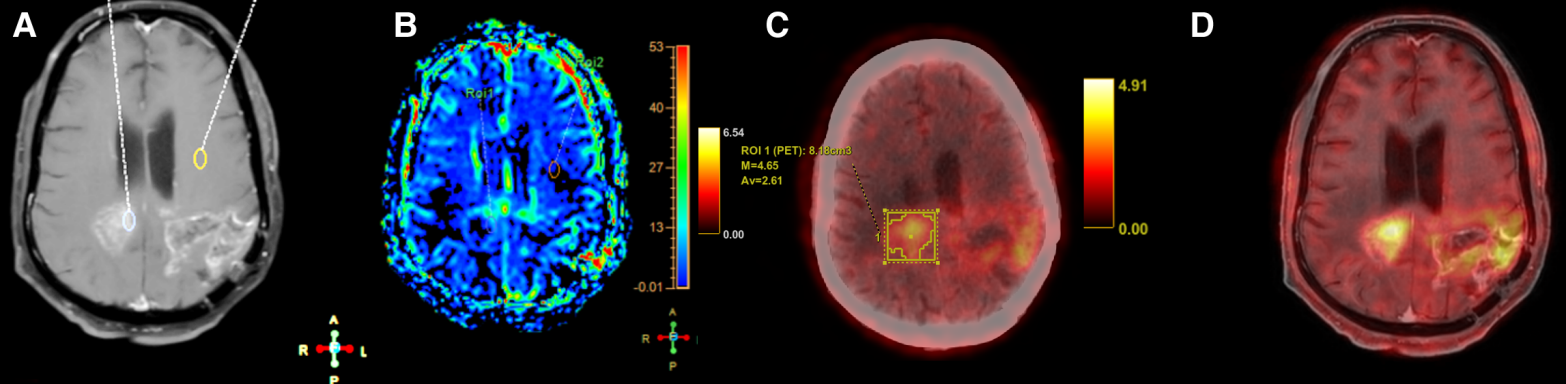
Axial T1 + Gd (**A**), perfusion map (**B**), FET-PET (**C**) and fused MRI and FET-PET images in a case of glioblastoma shows areas of thick nodular enhancement in bilateral posterior parietal lobes with hyperperfusion (**B**). FET-PET (**C**) shows significant uptake (T/W ratio: 3.6). Fused images (**D**) shows corresponding areas of increased perfusion and uptake, suggestive of recurrence.

**Figure 6:**
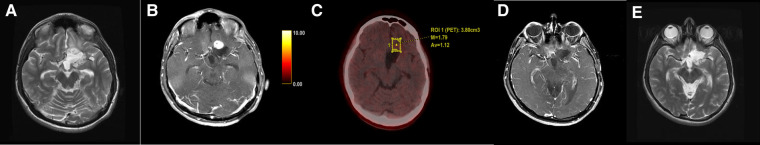
Axial T2-weighted (**A**) and T1 + Gd (**B**) images in a case of glioblastoma showed an enhancing nodule anterior to the post-operative cavity. FET-PET (**C**) showed no uptake in that area. Follow-up MRI (**D,E**) after 6 months showed complete resolution of the lesion.

Among 62 patients, 16 were classified as having radionecrosis, and 46 were finally classified as having recurrent brain tumors based on the outcomes criteria outlined in the earlier section [Figures 1–6].

The sensitivity, specificity, PPV, and NPV for determination of a tumor recurrence in a treated case of high-grade glioma with conventional MR imaging were 98.0%, 76.9%, 94.4%, and 90.9% respectively. 35 out of 46 cases of recurrence showed T2 intermediate to dark areas. 41 out of 46 cases of recurrence showed rCBV greater than 1.40. The most common enhancement pattern seen in recurrent lesions was solid nodular (18 cases), followed by thick rim enhancement (16 cases).

In case of FET PET CT, the sensitivity, specificity, PPV and NPV for determination of a tumor recurrence in a treated case of high-grade glioma were 78.8%, 84.6%, 95.3% and 50% respectively. The median T/Wm ratio was 3.1 (0–5.8), with an average T/Wm of 3.4 in cases of TR.

In cumulative analysis (MRI and FET PET CT combined), the sensitivity, specificity, PPV and NPV for determination of a tumor recurrence in a treated case of high-grade glioma was 97.9%, 100%, 100% and 91.6% respectively [Table 2].

**Table 2:** Comparison of sensitivity, specificity, PPV, NPV and accuracy.

Study	Sensitivity	Specificity	Positive predictive value	Negative predictive value	Accuracy
MRI	98.08%	76.92%	94.44%	90.91%	93.85%
					95% CI
					(84.99% to 98.30%)
FET PET CT	78.85%	84.62%	95.35%	50%	80.00%
					95% CI
					(68.23% to 88.90%)
Combined Studies	97.96	100%	100%	91.67	98.33%
					95% CI
					(91.06% to 99.96%

**Table 3:** Patient-wise data of the diagnostic process.

Case no.	Age	Histopathology	Interval from RT completion (in months)	rCBV	MRI diagnosis	T/Wm ratio	FET-PET diagnosis	Final diagnosis
1	47	Glioblastoma	21	0.47	Radiation necrosis	0.01	Radiation necrosis	Radiation necrosis
2	54	Glioblastoma	15	1.8	Recurrence	3.3	Recurrence	Recurrence
3	26	Glioblastoma	16	1.8	Recurrence	3.6	Recurrence	Recurrence
4	37	High-grade glioma	8	1.5	Recurrence	3.4	Recurrence	Recurrence
5	57	Glioblastoma	26	1.5	Recurrence	2.6	Recurrence	Recurrence
6	59	Glioblastoma	12	4.7	Recurrence	3.8	Recurrence	Recurrence
7	44	Glioblastoma	7	1.8	Recurrence	3.2	Recurrence	Recurrence
8	41	Glioblastoma	49	2.5	Recurrence	3.1	Recurrence	Recurrence
9	39	Glioblastoma	18	2.4	Recurrence	3.3	Recurrence	Recurrence
10	26	High-grade glioma	19	1	Recurrence	2.7	Recurrence	Recurrence
11	43	Glioblastoma	45	4.9	Recurrence	3.4	Recurrence	Recurrence
12	26	High-grade glioma	22	1.4	Recurrence	4.8	Recurrence	Recurrence
13	31	Glioblastoma	6	1.5	Recurrence	3.3	Recurrence	Recurrence
14	40	Glioblastoma	16	4.3	Recurrence	3.1	Recurrence	Recurrence
15	48	Anaplastic oligodendroglioma	46	1.6	Recurrence	3	Recurrence	Recurrence
16	35	Glioblastoma	22	3.5	Recurrence	5.5	Recurrence	Recurrence
17	50	Glioblastoma	18	2.5	Recurrence	3.5	Recurrence	Recurrence
18	49	Anaplastic astrocytoma	18	2	Recurrence	4	Recurrence	Recurrence
19	40	Glioblastoma	7	1.6	Recurrence	2.6	Recurrence	Recurrence
20	56	Anaplastic oligodendroglioma	8	2.6	Recurrence	4.2	Recurrence	Recurrence
21	45	Glioblastoma	58	2.8	Recurrence	3.5	Recurrence	Recurrence
22	58	Glioblastoma	17	1.75	Recurrence	2.6	Recurrence	Recurrence
23	46	High-grade glioma	84	3.4	Recurrence	3.6	Recurrence	Recurrence
24	72	Glioblastoma	6	2.2	Recurrence	3.5	Recurrence	Recurrence
25	54	Glioblastoma	15	4.8	Recurrence	3.6	Recurrence	Recurrence
26	56	Glioblastoma	27	1.43	Recurrence	2.9	Recurrence	Recurrence
27	49	Glioblastoma	13	3.4	Recurrence	3.6	Recurrence	Recurrence
28	44	High-grade glioma	23	1.3	Recurrence	4.1	Recurrence	Recurrence
29	32	High-grade glioma	32	2.09	Recurrence	4.56	Recurrence	Recurrence
30	58	Glioblastoma	25	1.6	Recurrence	3.5	Recurrence	Recurrence
31	37	Anaplastic astrocytoma	31	1.9	Recurrence	5.8	Recurrence	Recurrence
32	31	Glioblastoma	20	2.7	Recurrence	3.4	Recurrence	Recurrence
33	44	Glioblastoma	3	3	Recurrence	2.6	Recurrence	Radiation necrosis
34	50	Glioblastoma	10	2	Recurrence	5	Recurrence	Recurrence
35	56	Glioblastoma	10	11	Recurrence	4.8	Recurrence	Recurrence
36	25	Glioblastoma	19	2.8	Recurrence	2.8	Recurrence	Recurrence
37	33	Glioblastoma	15	1.9	Recurrence	3.5	Recurrence	Recurrence
38	28	Glioblastoma	26	2	Recurrence	3.7	Recurrence	Recurrence
39	50	Glioblastoma	11	2.8	Recurrence	3.4	Recurrence	Recurrence
40	57	Glioblastoma	18	2.2	Recurrence	3.6	Recurrence	Recurrence
41	72	Glioblastoma	11	1.8	Recurrence	3	Recurrence	Recurrence
42	41	Anaplastic oligodendroglioma	80	1.7	Recurrence	3.1	Recurrence	Recurrence
43	40	Glioblastoma	31	0.6	Recurrence	3.2	Recurrence	Recurrence
44	34	Anaplastic astrocytoma	16		Recurrence	2.7	Recurrence	Radiation necrosis
45	31	Glioblastoma	27	0.8	Radiation necrosis	3.08	Recurrence	Recurrence
46	74	Glioblastoma	8	2	Recurrence	2.3	Radiation necrosis	Recurrence
47	32	Glioblastoma	4	2	Recurrence	1.9	Radiation necrosis	Radiation necrosis
48	47	Glioblastoma	24	1.7	Recurrence	2.2	Radiation necrosis	Radiation necrosis
49	43	Glioblastoma	24	0.8	Recurrence	2.2	Radiation necrosis	Recurrence
50	32	Glioblastoma	13	1.7	Recurrence	1.9	Radiation necrosis	Recurrence
51	51	Glioblastoma	22	1.5	Recurrence	2.1	Radiation necrosis	Radiation necrosis
52	40	Anaplastic astrocytoma	73	1.8	Recurrence	0.01	Radiation necrosis	Recurrence
53	36	Anaplastic oligodendroglioma	67	1.47	Recurrence	1.8	Radiation necrosis	Radiation necrosis
54	38	Anaplastic oligodendroglioma	21	0.9	Radiation necrosis	1.5	Radiation necrosis	Radiation necrosis
55	42	Anaplastic astrocytoma	15	0	Radiation necrosis	1.8	Radiation necrosis	Radiation necrosis
56	49	Glioblastoma	29	0.87	Radiation necrosis	2.2	Radiation necrosis	Radiation necrosis
57	35	Anaplastic astrocytoma	15	0	Radiation necrosis	2.2	Radiation necrosis	Radiation necrosis
58	47	Glioblastoma	2	1.08	Radiation necrosis	1.9	Radiation necrosis	Radiation necrosis
59	63	Glioblastoma	15	0	Radiation necrosis	1.6	Radiation necrosis	Radiation necrosis
60	63	Glioblastoma	24	0.8	Radiation necrosis	1.6	Radiation necrosis	Radiation necrosis
61	36	Anaplastic oligodendroglioma	4	0.48	Radiation necrosis	0	Radiation necrosis	Radiation necrosis
62	48	Anaplastic oligodendroglioma	63	1.3	Radiation necrosis	2.4	Radiation necrosis	Radiation necrosis

In 4 cases, MRI yielded a false positive diagnosis. Out of these 4 cases, FET-PET yielded a true negative diagnosis in two cases and false positive diagnosis in the other two cases. In 4 cases, FET-PET yielded a false negative diagnosis. In one case of Li Fraumeni syndrome with GBM and metastatic breast cancer, MRI yielded a false negative result.

## Discussion

Routine diagnosis and treatment monitoring of brain tumors is usually based on contrast-enhanced MRI. Radionecrosis is a consequence of radiation injury to normal brain tissue, which results in peritumoral white matter necrosis and endothelial cell dysfunction (33). This manifestation usually occurs between 6 months and 2 years after the completion of radiotherapy (11). Although in a proportion of patients, RN will be asymptomatic on surveillance imaging, symptoms may include features of raised intracranial pressure, worsening of neurological deficits, or new onset seizures. The symptoms and timing of symptoms are indistinguishable from that of tumor progression. Therefore, clinicians require standardized neuroimaging of these lesions to help establish a diagnosis and potentially guide therapy. The gold standard for differentiating tumor recurrence from radiation necrosis remains histopathological evaluation. However, tissue diagnosis is an invasive procedure associated with the risk of major complications.

Conventional MRI, along with advanced sequences like diffusion-weighted imaging, perfusion-weighted imaging, and MRS are often considered the standard of care for estimation of treatment response and surveillance following treatment completion for gliomas (34–36). However, early phases during evolving necrosis or recurrence can present with similar features on MRI, showing contrast enhancement, mass effect, and vasogenic edema (37). FET-PET utilizes the preferential uptake of radiolabeled amino acid tracers by tumor cells to produce an enhanced tumor-to-background contrast. This has proved useful in the differentiation of TR from RN, as well as various other indications like monitoring of treatment, prognosis, or grading of glioma. Our study demonstrated the best results using both MRI and FET-PET in conjunction, with an accuracy of 98.3% to detect TR.

On T2-weighted imaging, radiation necrosis is seen as a central necrotic component with increased signal intensity (SI), while the peripheral portion is seen as low SI. Perilesional edema is commonly seen (37). Recurrent disease, meanwhile, is seen as T2 intermediate to dark signal intensity with similar imaging features as the known primary tumor. Most viable tumor sites are highly cellular tissues containing large amounts of membranes and macromolecules; the highly cellular component of cerebral glioma would exhibit less hyperintensity on T2WI (38). Schwartz et al. demonstrated that lesions with a hypointense arc on T2 and a heterogeneous center are likely a neoplasm, either glioma or metastasis. Another study by Dequesada et al. described a feature called lesion quotient, which was defined as the proportional value of the maximum cross-sectional area of a nodule with distinct borders (on the T2-weighted sequence) with the enhancing area on the T1-weighted post gadolinium sequence on a comparable axial section (39). It had high predictive value, sensitivity, and specificity for identifying the presence of radiation necrosis alone (39). It is a well-known fact that cellular tumors are hypointense on T2-weighted images. The lesion quotient is the proportion of T2 hypo intensity within the enhancing nodule and can help in differentiating tumor recurrence from radiation necrosis (40). Our study concurs with the findings demonstrating T2 intermediate to dark areas in 35 out of 46 cases of tumor recurrence. The T1-relaxation shortening on the passage of the contrast agent after intravenous injection of contrast causes a signal increase in T1-weighted MRI, causing better tissue delineation between diseased or injured tissues, also called dynamic contrast-enhanced (DCE) MRI (30). The commonly seen enhancement patterns in radiation necrosis are “soap-bubble-like,” “swiss-cheese-like,” and “cut green pepper”. Swiss cheese lesions result from diffuse necrosis affecting the white matter and cortex with a diffuse enhancement of feathery margins and intermixed necrotic foci (37).

The metabolic signatures of brain tumors have been extensively studied using single and multivoxel proton MRS techniques. Several studies have revealed that in tumor tissue, choline levels are higher while N-acetyl-aspartate (NAA) levels are lower than they would be in normal brain parenchyma. Certain tumors, such as high-grade gliomas, frequently exhibit resonance related to lactate or lipids. During radiation therapy, proton-MRS of brain tumors has been demonstrated to be helpful for detecting recurrence. Previous studies have discussed the clinical utility of proton MRS using the Cho/Cr ratio to distinguish residual/recurrent glioblastoma from necrosis (41).

Dynamic susceptibility-weighted contrast-enhanced MRI is a T2*-weighted technique to measure relative cerebral blood volume (rCBV), which allows for measurements of the vascular environment surrounding a tumor. Several studies have shown different mean rCBV cutoff values of 0.71(2009) (42), 1.49 (2011) (43), and 1.75 (2011) (44) to distinguish radiation necrosis from tumor recurrence reliably. Law et al. attained 95.0% sensitivity and 57.5% specificity at 1.75 as the threshold value (2003) (45). Shin et al. calculated a cut-off value of 2.93 for the rCBV ratio sensitivity 90.9%, specificity 83.3% (46). In this study, a cutoff of 1.48 was calculated using ROC curve analysis. Two cases with raised rCBV turned out to be radiation necrosis. The possible reason for the same is the ROI of rCBV being kept at a hemorrhagic area within the treatment site. The areas of hemorrhage have also contributed to false positive findings in FET-PET.

[18F]-2-Fluoro-2-deoxy-D-glucose (FDG) is the most commonly used metabolite in most of the PET scans. However, due to the lower contrast of lesions from the normal cerebral cortex, the use of FDG-PET scans is often challenging in routine neuro-oncology (30, 31). The cerebral uptake of radiolabeled amino acids is usually low, but it is typically increased in brain tumors. This produces an enhanced tumor-to-background contrast. Also, the uptake of amino acid tracers is independent of the integrity of the blood-brain barrier, hence the evaluation of non-enhancing gliomas can be done with amino acid PET. The ability of amino acid PET to distinguish between changes caused by the treatment and tumour progression has also been established. Additionally, it is used for a number of other purposes, including grading gliomas and evaluating therapy progress. The radiotracer O-[2-(18F)-fluoroethyl]-L-tyrosine (FET) has emerged as the most used amino acid tracer for the diagnosis of brain tumours. A few more are MET and FDOPA (31, 47). The amino acid transport system L for large neutral amino acids, namely the subtypes LAT1 and LAT2, are responsible for the increased uptake of MET, FET and FDOPA in gliomas and brain metastases (30, 31). A feature that distinguishes FET from MET and FDOPA is the high metabolic stability of FET, as it has been demonstrated that MET and FDOPA undergo some metabolism and are incorporated into protein, whereas FET is not metabolized. Furthermore, studies have shown that over-expression of LAT1 is closely associated with a malignant phenotype and proliferation of gliomas (31). Our study utilized a T/Wm cutoff of 2.65 in our analysis with T/Wm >2.65 suggestive of TR and T/Wm <2.65 suggestive of RN. This was based on prior studies that showed a cutoff of 2.65 yielded a sensitivity of 80% and specificity of 87.5% (32). Tumor recurrence or radiation necrosis is always associated with inflammation. There have been instances of non-specific focal 18F-FET and 11C-MET uptake near hematomas, cerebral ischemia, brain abscesses, acute inflammatory demyelination, sarcoidosis, and radiation necrosis. Additionally, reactive astrocytosis is seen around brain tumours and radiation therapy is known to trigger astrogliosis. The efficacy of this approach for surgical resection and radiation therapy planning in recurrent gliomas may depend critically on whether increased 18F-FET-uptake in such regions causes an overestimation of tumour size. However, 18F-FET uptake in the region of experimental gliomas in the form of astrogliosis was only marginally more than that seen in human peritumoral tissue (48–53).

Static PET imaging provides a single snapshot of radiopharmaceutical concentration. On the other hand, dynamic PET consists of acquiring sequential series of images as the tracer distributes in the tissues, followed by kinetic curve analysis to provide parametric images. Static 18F-FET scans have shown to have higher accuracy for glioma grading than dynamic scans (54). Moreover, dynamic FET-PET imaging is a time-intensive process and was deemed to be less feasible given the constraints of a busy institute such as ours.

The true diagnostic positive rate of MRI (sensitivity) was 98.08%, and the true diagnostic negative rate (specificity) was 76.92% in this series. These favorable results of MRI should be considered carefully, as they might be biased by the retrospective study design: all patients with suspected tumor recurrence were considered for PET. Rachinger et al., compared FET-PET with MRI involving 46 patients; the sensitivity and specificity of FET-PET for the detection of recurrent tumors were 100% and 93%, respectively, compared with 93% and 50% for MRI (55). A recent systematic review and meta-analysis on the discriminators of pseudoprogression and true progression in high-grade gliomas demonstrated that dynamic susceptibility contrast perfusion MRI (DSC-MRI) and DWI showed the highest diagnostic accuracy (56). In our study, the lower specificity of MRI compared to FET-PET can be attributed to a selection bias as FET-PET was ordered only for patients with equivocal findings on MRI. As FET-PET was not performed in cases with unequivocal progression on MRI, overall specificity of MRI was lowered. In this study, the gold standard used was either surgical excision or stereotactic biopsy.

Studies have utilized simultaneous FDG-PET/MR imaging to evaluate the diagnostic performance of functional MR imaging and PET parameters when used individually and in combination and have concluded that these add synergistic benefits when utilized together. Parameters like rCBVmean (mean relative CBV), ADCmean, Cho/Cr, and maximum and mean target-to-background ratios were statistically significant in the detection of recurrent lesions with an accuracy of 77.5%, 78.0%, 90.9%, 87.8%, and 87.8%, respectively, and a maximum AUC was achieved by combining FDG and MRI parameters (57).

For imaging these lesions, it is important to remember that certain diagnostic tests may be more sensitive or specific than other more conventional tests. The principal basis of most imaging is to differentiate metabolically active tissues by analyzing the cell-specific uptake in malignant tissue, which would always be higher than the metabolic activity of necrotic tissue. An imaging modality that incorporates the most discrimination (highest specificity) may be the most reliable test for distinguishing TP from RN. In our study, both MRI and FET PET have comparable specificity, however, this might be due to the fact that the number of cases with true negatives was less.

In contemporary clinical practice, the response assessment for high-grade gliomas are guided by the Response Assessment in Neuro-Oncology (RANO) criteria (58–60). The importance of detecting early recurrences with good accuracy is important for clinical practice as well as for deciding on patients eligible for clinical trials addressing recurrent glioma. In an ideal situation, it would be more prudent to identify early true recurrences since small volume recurrences might be amenable for effective salvage treatments, and early initiation of therapy might improve survival. As a corollary, detection of RN avoids overtreatment, reducing the morbidity of aggressive treatment regimens, and can guide the initiation of steroids to decrease the inflammatory process at play in RN. One of the major limitations of RANO imaging-based assessment is the use of morphological assessment using conventional MRI sequences, which can be challenging to differentiate RN from progression in a substantial number of patients. In clinical practice, it will be appropriate to consider MRI as the preferred modality for routine surveillance and use FET-PET in conjunction where MRI findings are equivocal. Also, we suggest the utilization of advanced imaging techniques whenever available, which can provide insights into the underlying pathological alterations. Also, better reproducibility such as rCBV (perfusion MRI), and Choline: NAA ratio (MRS), can mitigate the subjective variations in the interpretation of morphological features alone.

The study was performed in a small but uniform group with a short interval between the two imaging modalities. All cases were discussed individually on a multidisciplinary tumor board which added further robustness to our data, however, there was limited availability of histopathological evidence in the majority. Furthermore, because only patients with equivocal MRI findings and limited therapy choices were referred to FET-PET imaging, it is likely biased towards challenging cases. The role of the administration of steroids is unclear, as it was initiated in many cases following the MRI, and this could have possibly reduced the inflammatory changes in cases of RN, thereby allowing for better interpretation of FET-PET imaging. While the use of MRS and perfusion imaging along with conventional MRI further improved results, newer techniques such as arterial spin labeling (ASL) MR perfusion also need to be studied in this context. Further studies with larger sample sizes, as well as the development of an MRI-based scoring system, are being pursued to develop a robust and reproducible model.

## Conclusions

Our findings support the use of MRI and [18F]FET PET in combination to distinguish RN from recurrence in gliomas with excellent accuracy. To improve clinical decision-making, we propose a stepwise approach as a resource-saving and cost-effective strategy with regular MRI-based surveillance and using FET-PET in conjunction for patients with equivocal MRI findings.

## Data Availability

The raw data supporting the conclusions of this article will be made available by the authors, without undue reservation.
